# Field study demonstrates inordinate respirable dust generation during continuous mining in rock versus coal strata

**DOI:** 10.1007/s40789-025-00832-y

**Published:** 2025-11-10

**Authors:** F. Animah, E. Sarver

**Affiliations:** Department of Mining and Minerals Engineering, Virginia Polytechnic Institute & State University, Blacksburg, VA USA

**Keywords:** Respirable coal mine dust, Respirable silica, Dust control, Dust scrubber, Water sprays

## Abstract

In modern room and pillar coal mines, the coal is produced by continuous miner (CM) machines. The CM is used to mine the coal seam by continuously cutting at a vertical face. Depending on the seam thickness, quality, and geotechnical properties, some roof, floor, or interburden rock is often cut along with coal. While CMs can be highly efficient in terms of production rates, they can also generate high concentrations of dust. Dust poses both safety (i.e., explosibility) and respiratory health hazards. Previous research has generally indicated that CM cutting in rock yields much more respirable dust than cutting in coal. Although in-mine studies that directly evaluate this trend have not been reported, understanding relative dust generation from different geologic strata could have important implications. In many mines, for instance, the rock is the primary source of respirable silica and silicates, which can be especially hazardous. To mitigate dust generated by the CM, mines use a variety of controls including ventilation, on-board scrubber systems, and water sprays. However, the relative effects of controls on dust generated from different strata have also not been widely investigated. In this field study, respirable dust sampling was conducted in the intake and return airways of an active CM during periods when the cutting was targeted either primarily at the coal seam (bottom cut) or primarily at the roof rock (top cut) in a standard entry. Results indicated that CM cutting in rock strata generated somewhat finer particles and respirable dust concentrations that were 2.1–26 times higher than cutting in coal strata, although the coal height being cut was about 2.2-2.9 times greater than the rock height. Additionally, the analysis of dust mineralogy generally showed a mix of both carbonaceous (coal) and mineral particles regardless of the target strata. Furthermore, the study was designed to evaluate the effects of two typical combinations of CM scrubber and ventilation conditions, and increased pressure and volume through the CM water sprays. In general, operation of the scrubber tended to yield lower and somewhat finer respirable dust concentrations, irrespective of the strata the CM was targeting. Increased water spray pressure and volume sometimes appeared to reduce the respirable dust concentration when the CM was targeting the roof rock, but no effect could be discerned when the CM was targeting the coal seam.

## Introduction

Room and pillar mining is one of the most common methods used to produce coal in underground operations. In modern operations, continuous miner machines (CMs) are typically used to cut the coal at a vertical face, advancing a grid of entries (i.e., rooms) and crosscuts between them, and leaving pillars in place as roof support (Michalski 2011). CMs are a major source of dust in underground coal mines (Colinet et al. 2013; NIOSH 2021). The respirable fraction of the dust presents a health hazard, especially if it contains respirable crystalline silica (RCS) (Cohen 2010; Cohen et al. 2016, 2022; Hall et al. 2019, 2024; National Academies of Sciences, Engineering, and Medicine 2018). Due to the quality and geotechnical properties of the coal seam and surrounding rock strata, and considerations of equipment sizes and ergonomics, some rock (roof, floor, or interburden) is often cut along with the coal. In many mines, the rock strata is the primary source of silica (Keles and Sarver 2022; Schatzel 2009) and therefore, mines cutting substantial rock are more prone to high RCS concentrations (Beck et al. 2016). Moreover, there is at least some evidence that RCS is more concentrated in the finer size fractions of dust generated from cutting rock (Organiscak et al. 1990; Ramani et al. 1987), which could further impact the relative health hazard.

Moreover, prior studies by the authors’ research group have indicated that CM cutting into rock strata generates substantially more respirable dust than CM cutting into the coal seam itself (Jaramillo et al. 2022; Sarver et al. 2021). Using data from 18 different mines, Jaramillo et al. (2022) conducted a simple source apportionment exercise based on a comparison of the composition of respirable dust samples collected just downwind an active CM and the composition of the raw coal and rock strata materials being cut by the CM at the face. On average, while the rock strata represented 37% of the total mining height (i.e., the other 63% of the height was in coal), it contributed 71% of the respirable dust generated by the CM. However, field studies have not been reported enable direct evaluation of the concentration and characteristics of respirable dust generated from rock versus coal strata.

Likewise, there is limited data surrounding the effects of specific controls on dust sourced from different strata. In CM operations, there are typically three main types of controls used to mitigate dust around the production face. Ventilation is the most ubiquitous of these (Xu et al. 1991). At the CM face, either blowing or exhausting ventilation schemes often involve the use of a line curtain to efficiently direct dust (and liberated gas) toward the return air course and prevent recirculation back into the intake where workers are positioned (Mohamed et al. 1996; Organiscak and Beck 2010). In some cases, ventilation tubing is also used for additional dust control in CM sections (Potts et al. 2011).

CM machine-mounted flooded-bed scrubbers (FBS) are a second type of control, and they serve to capture dust generated at the production face (Organiscak and Beck 2010). FBSs operate by drawing dust-laden air through a wet filter screen, which removes some particles outright. This causes other dust particles to be taken up by water droplets that can then be captured in a demister before the cleaned air exits via the scrubber exhaust (Colinet and Jankowski 2000; NIOSH 2021; Organiscak and Beck 2010). Several studies have looked at the relative efficiency of FBSs on particles of different sizes and types. Previous research by the authors generally found that scrubber efficiency is particle size-dependent, although no appreciable differences could be observed in the removal efficiency by particle type (Animah et al. 2023, 2024). However, Colinet et al. (1990) investigated scrubber efficiency on different particle types in a series of laboratory experiements and results indicated higher efficiencies for respirable-sized coal versus quartz (i.e., which represents the primary form of respirable crystalline silica in most coal mines). Nevertheless, the effect of the scrubber on dust generated from specific strata has not been directly tested.

Water sprays are the third main type of dust control applied in CM operations. Sprays are typically mounted on the CM, with a variety of different nozzle designs and configurations devised to create a fine mist that suppresses, captures, and redirects airborne dust particles at the point of generation (Kissell 2003; Potts et al. 2011). For example, sprays on the CM drum help to wet the face ahead of and during mining (dust suppression). Also, sprays on the CM chassis and throat can capture airborne dust, while sprays on the sides of the CM body can block dust movement toward the operator. Aside from these, some CMs (called ‘wetheads’) have water sprays built into the cutting bits on the shearer drum. The bit sprays are designed for cooling (i.e., to prevent sparking), but can also enhance dust suppression (Chugh et al. 2006; Goodman et al. 2006; Listak et al. 2010). There have been numerous investigations into spray optimization for dust control, including the impact of increased water pressure and volume, but overall results have been mixed (Beck et al. 2018; Colinet et al. 1991; Jayaraman et al. 1984; Jiang et al. 2022, 2023; Pollock and Organiscak 2007; Schroeder et al. 1986). This might be due to variable field conditions, for instance, as related to CM operational parameters (e.g., power, drum rotational speed, bit wear) or the properties of the mine strata being cut by the CM. These variable field conditions could influence the concentrations, sizes, and types of dust being generated. In general, it is well established that sprays are less effective on respirable dust (i.e., less than about 10 μm) than on coarser fractions (Cheng et al. 1974; Organiscak et al. 1990; Xu et al. 1991), and the finest dust particles could even be re-aerosolized by the local turbulence induced by water sprays (Jiang et al. 2022, 2023). Indeed, micron-scale dust particles are much less likely than coarser particles to be captured by water droplets from typical sprays. This is due to the relatively large difference in size between the particles and droplets. For better capture of fine dust particles, Colinet (2021) found that sprays that generate smaller water droplets such as full-cone and flat-fan sprays should be employed on the CM. Additionally, these sprays can be more efficient on finer particles when positioned closer to the dust source and by also adding surfactants in the water sprays to enhance their wettability (Colinet et al. 2010).

To fill some of the knowledge gaps highlighted above, this field study was conducted to evaluate the relative differences in respirable dust concentration and characteristics (particle size and mineralogy) generated from the CM cutting in coal versus rock strata. This was accomplished by altering the standard procedure for a full CM cut, which typically involves cutting the entire height of the entry. Instead, it was modified to include two consecutive cuts: the first cut targets the coal seam (referred to as the bottom cut), and the second cut focuses on the roof rock (known as the top cut). This approach allows for the dust generated from each cut to be sampled separately. The ‘bottom, then top’ cutting practice was repeated in a series of tests that also enabled the evaluation of the effects of (1) two typical FBS and ventilation conditions, and (2) increased pressure and volume through the CM water sprays.

## Materials and methods

### Mine details

The study was conducted in an underground room and pillar mine in central Appalachia (termed ‘Mine 27’ from here). Mine 27 produces bituminous coal from the Pocahontas No. 3 seam in four separate sections. The average seam thickness across the entire mine is about 1.3 m and the total mining height is typically 1.8–2.0 m. Rock in the mine usually consists of shale and sandy shale and is generally cut from the roof with limited extraction from the floor. For this study, sampling was only conducted in Sects. 1 and 2, which are both 9-entry super sections. On each side of a super section, the mine runs a CM, twin-boom roof bolter, and two shuttle cars. The CMs had a total machine power of around 610 kW (820 HP). The cutter head diameter was between 0.97 and 1.1 m. Sampling was conducted on non-production shifts, and there was no bolting upwind of the CM. Ventilation in each section is split into the 9 entries with the intakes in the central entries and returns at the last two left and right entries. At the time of sampling, Mine 27’s approved ventilation plan called for exhausting ventilation with a line curtain and *without* the FBS operating for flush cuts (i.e., shallow depth into the heading); and for standard cuts (i.e., further into the heading), the plan called for blowing ventilation with line curtain and the FBS operating. All CMs at Mine 27 are equipped with an FBS (operating at air quantities of 3.5 m^3^/s (7500 CFM)–3.6 m^3^/s (7700 CFM), and at 30HP) and CM-body and drum water sprays, and most CMs (including those operated for this study) have wetheads. The mine’s ventilation plan specifies a minimum spray pressure and volume that must be used for both the bit sprays and all other sprays.

### Dust sampling

**Fig. 1:**
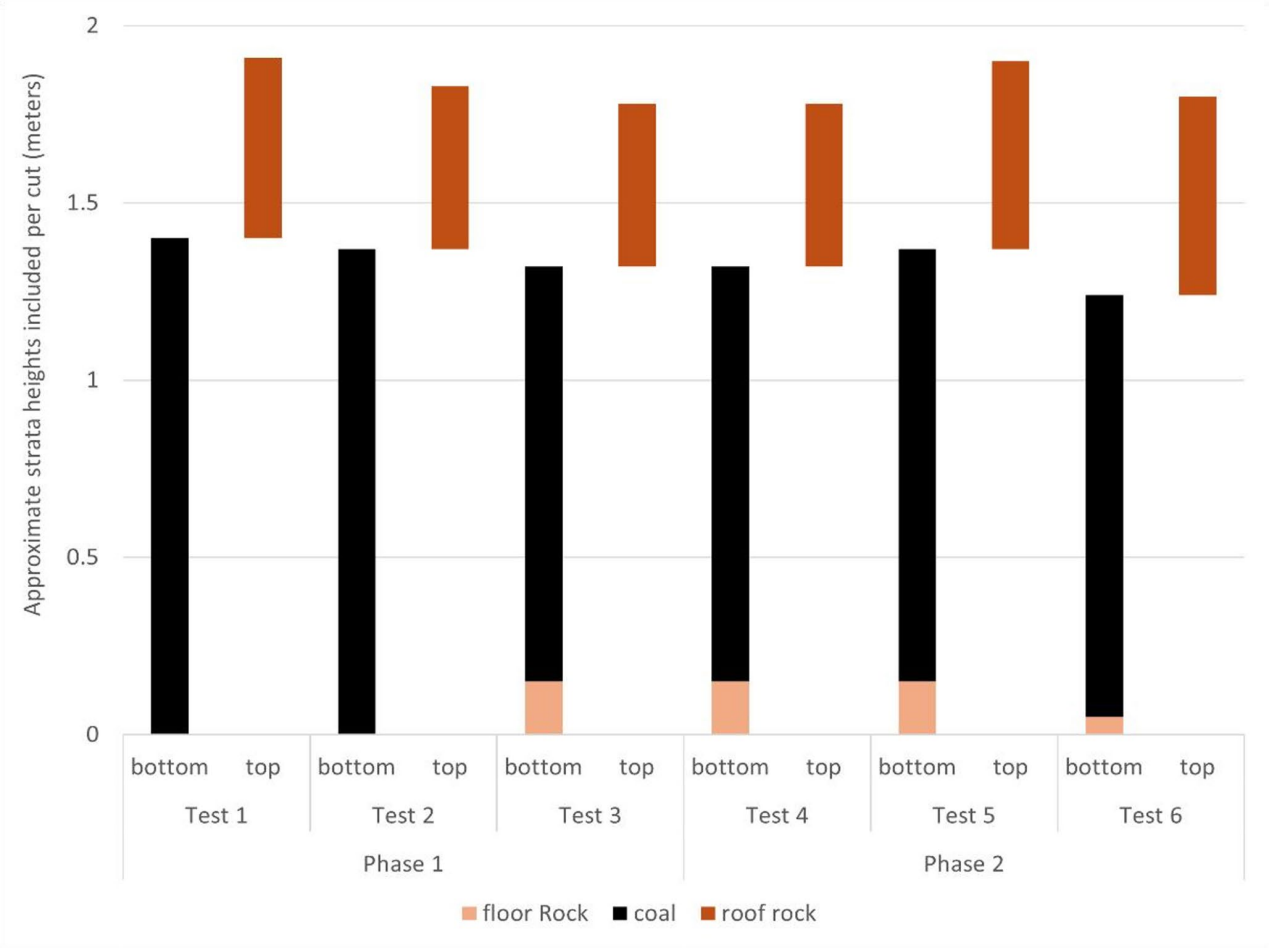
Approximate strata heights included in the bottom and top cuts during each test. The sum of the bottom and top cut heights is the total mining height in the entry

In total, six different tests were conducted, with each test including ‘bottom, then top’ cuts under two different FBS or water spray conditions (Table 1). Figure 1 shows the relative heights for the bottom versus the top cut in each test. As mentioned, the CM operator targeted the coal seam for the bottom cut and the typical extent of roof rock extraction for the top cut. (Notably, care was taken to maintain similar CM power between bottom and top cuts.) As shown, measurable bottom rock was cut with the coal in tests 3–6 (common in Mine 27), and a limited amount of bottom rock may have also been taken in tests 1 and 2. The separation between the bottom and top cuts was based on the visual interface between the coal and roof rock and the CM operator’s experience in the ‘feel’ of the two strata (i.e., the rock is generally harder and more resistant to cutting). However, it must be acknowledged that the interface is imperfect, and the sheer size and maneuverability of the CM drum also limit the ability for precision cutting. Notably, for this study, the same operator performed all CM cuts, and he was highly experienced.

**Table 1:** Summary of test conditions in phase 1 and phase 2 tests

Phase	Test					Cut			
	No.	Location	Airflow (m³/s)	Control Variable	Condition	Sequence	Height (m)	Depth (m)	Time (mins)
1	1a	Section 2 entry 7	7.8	FBS/vent	off/exhausting	bottom	1.40	4.57	13
						top	0.51		12
	1b		4.3	FBS/vent	on/blowing	bottom	1.40		15
						top	0.51		13
	2a	Section 2 entry 8	8.4	FBS/vent	off/exhausting	bottom	1.37	3.05	23
						top	0.46		14
	2b		not measured	FBS/vent	on/blowing	bottom	1.37		12
						top	0.46		16
	3a	Section 1 entry 7	7.9	FBS/vent	off/exhausting	bottom	1.32	3.05	6
						top	0.46		12
	3b		4.3	FBS/vent	on/blowing	bottom	1.32		13
						top	0.46		18
2	4a	Section 1 entry 8	7.9	CM sprays	min *P*&*V*	bottom	1.32	3.05	23
						top	0.46		23
	4b			CM sprays	max *P*&*V*	bottom	1.32		12
						top	0.46		15
	5a	Section 1 entry 2	8.0	CM sprays	min *P*&*V*	bottom	1.37	3.05	17
						top	0.53		12
	5b			CM sprays	max *P*&*V*	bottom	1.37		11
						top	0.53		10
	6a	Section 1 entry 3	7.9	CM sprays	min *P* + *V*	bottom	1.25	3.05	18
						top	0.56		10
	6b			CM sprays	max *P*&*V*	bottom	1.25		17
						top	0.56		10

Note: ‘on’ means when the CM scrubber was operating and ‘off’ means when the CM scrubber was not operating. ‘min *P* + *V*’ means when CM sprays were operating at standard pressure and volume of water conditions, and ‘max *P* + *V*’ means when the CM sprays were operating at the maximum pressure and volume of water)

Per Table 1, the first three tests were considered Phase 1 and were aimed at evaluating the dust generated from each stratum (coal or rock) under typical FBS and ventilation conditions specified for flush cuts (i.e., FBS off, exhausting ventilation) versus the conditions specified for standard cuts (i.e., FBS on, blowing ventilation). The Phase 1 tests always started with the CM cutting a flush heading, such that the entire test could be conducted within a single entry (i.e., to minimize variation in the strata heights and composition). During the Phase 1 tests, all CM sprays were operated with the minimum water pressure and volume specified in the mine’s ventilation plan. The final three tests were considered Phase 2 and were aimed at evaluating the effect of the CM’s water spray pressure and volume under typical conditions (i.e., at the minimum levels specified in the ventilation plan) versus maximum achievable pressure and volume (Table 2). All tests in Phase 2 were conducted during flush cuts (i.e., with the FBS off and exhausting ventilation conditions). Before each test (and for each ventilation condition in Phase 1 tests), the airflow across the CM was estimated (i.e., based on measured velocity and airway dimensions).

**Table 2:** Summary of the CM spray pressure and volume for phase 2 testing

Test	Location	Spray type	Minimum		Maximum	
			Pressure (kPa)	Volume (L/min)	Pressure (kPa)	Volume (L/min)
4	Section 1 entry 8	bit sprays	414	2.18	524	2.55
		other sprays	552	2.86	655	3.09
5	Section 1 entry 2	bit sprays	414	2.18	621	2.82
		other sprays	565	2.86	724	3.27
6	Section 1 entry 3	bit sprays	414	2.18	621	2.82
		other sprays	565	2.86	724	3.27

**Fig. 2:**
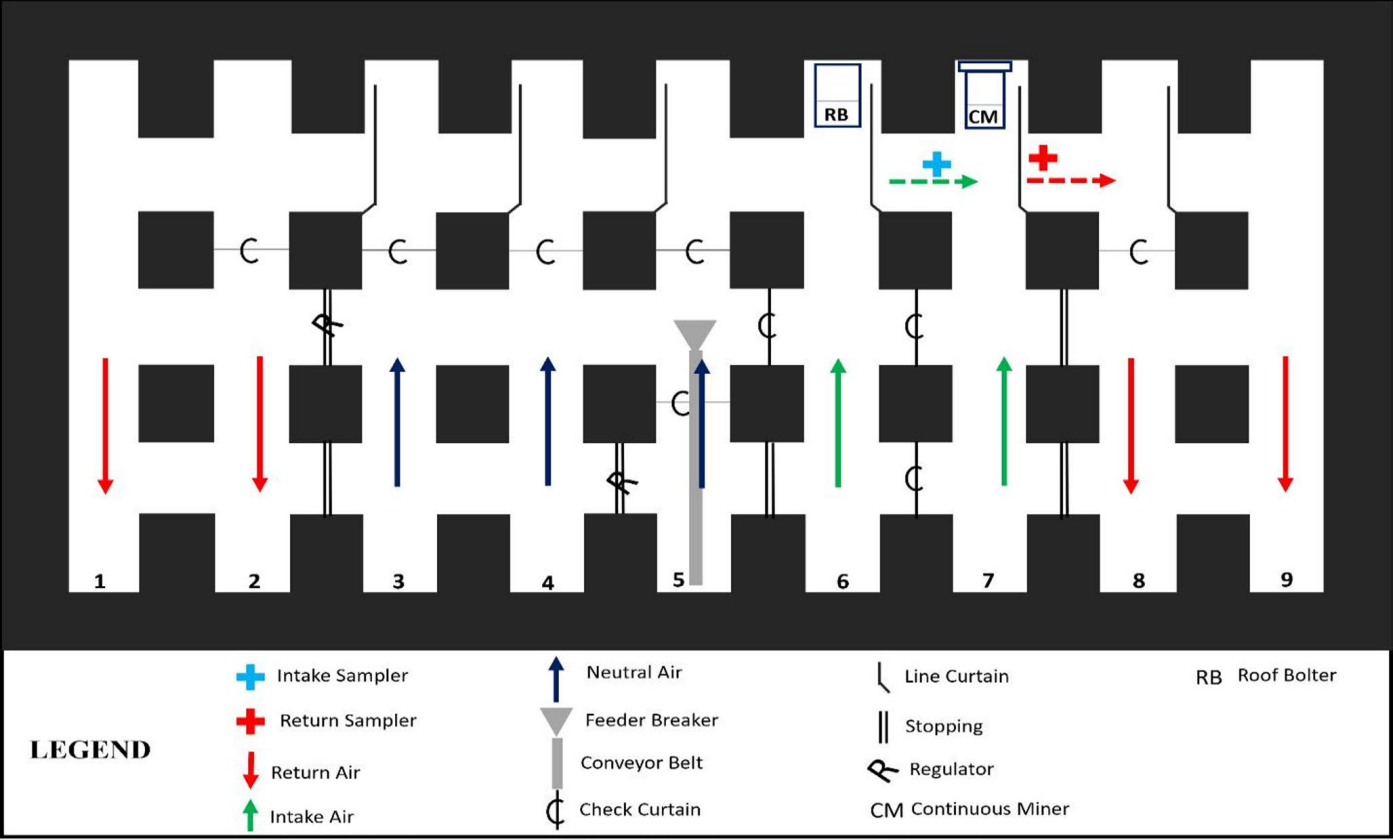
Schematic of ventilation and some select sampling locations in Mine 27

Sampling was conducted during each of the 24 total cuts shown in Table 1. For each cut, samples were collected in the CM intake and return (Fig. 2) over the entire cutting time. In both locations, standard Coal Mine Dust Personal Sampling Units (CMDPSUs) were used to collect sets of filter samples. Each CMDPSU consisted of a 10-mm Dorr Oliver nylon cyclone and an Escort ELF air pump operating at 2 L/min. (Zefon International, Ocala, FL) to collect just the respirable fraction of airborne dust directly onto a 37-mm filter housed in a 2-piece cassette. Each set of samples included three polycarbonate filters (PC, track-etched, 0.4 μm pore size). These were analyzed by scanning electron microscope to characterize particle size and mineralogy distributions (see below).

While mass concentrations of respirable dust can also be measured with CMDPSU sampling, this requires the collection of sufficient sample mass for gravimetric analysis. The sampling design here was not conducive to this approach given the relatively short cutting times (i.e., typically between 10 and 20 min). Thus, an alternative approach was taken. In both the CM intake and return locations, an additional CMDPSU was paired with a Personal data RAM (pDR-1000; Thermo Fisher, Waltham, MA), and these two samplers were run for relatively long total sampling periods (i.e., about 5 h on each day of the study). The CMDPSU collected dust onto a pre-weighed polyvinyl chloride filter (PVC, 5 μm pore size), which was used for gravimetric analysis to determine the time-weighted average (TWA) mass concentration of dust for the entire sampling period (see below). The pDR was used to log time series data (on a 30-s interval) on particle concentrations in the respirable size range, which can be calibrated to mass concentration based on the TWA concentration derived from the CMDPSU filter sample. This enabled mass concentrations to be determined for the intake and return locations during the time increments corresponding to each CM cut.

### Dust analysis

**Fig. 3:**
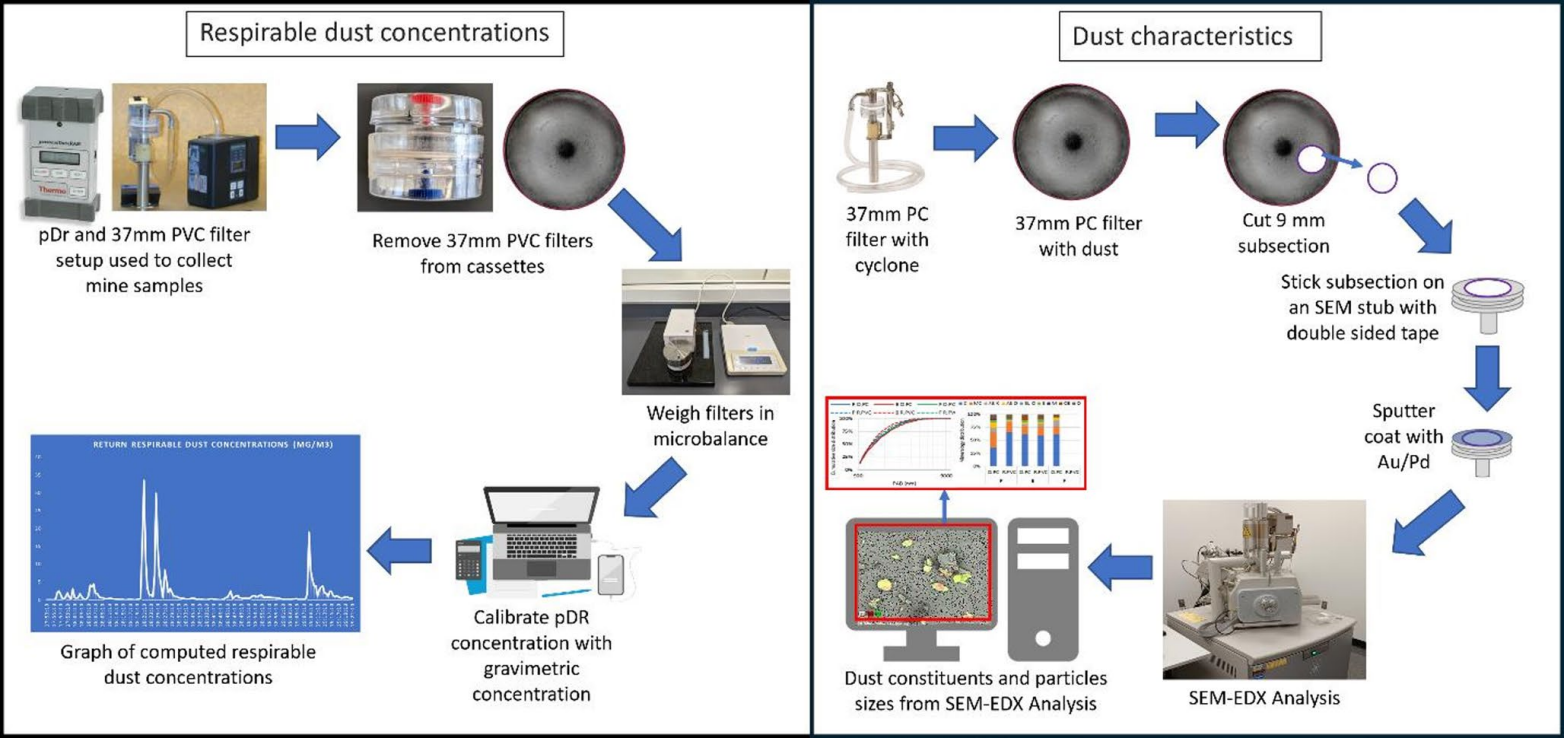
An illustration of the procedure used to obtain respirable dust concentrations and dust characteristics from collected samples in the study

Figure 3 summarizes the dust analysis for this study. To determine respirable dust mass concentration in the CM intake and return locations for each cut, the pDR data from the respective location and sampling time increment were interpreted. Briefly, the PVC filter samples collected alongside each pDR were post-weighed using the same microbalance used to measure filter pre-weights (Sartorius MSE6.6 S, Gottingen, Germany), and then, the TWA respirable dust mass concentration for each filter (*C*_filter_) was calculated by Eq. (1):

1 $${C_\text{filter}} = \frac{{{W_2} - {W_1}}}{{t \times f}}*100\,{\text{mg}}$$

where *W*_1_ and *W*_2_ are the PVC filter pre- and post-weight, respectively; *t* is the total PVC filter sampling duration; and *f* is the sampling flow rate.

Then, the ratio of filter sample TWA respirable dust mass concentration to pDR TWA particle concentration was determined as a calibration factor (*R*) for each pDR data set per Eq. (2):

2 $$R = \frac{{{C_\text{filter}}}}{{{C_\text{pDR}}}}$$

where R is the calibration factor; and *C*_pDR_ is the TWA particle concentration measured by the specific pDR unit paired with the filter sample from which *C*_filter_ was determined.

The calibration factor was applied to each pDR dataset to transform the time series particle concentration values to respirable dust mass concentration values. Finally, the concentration values corresponding to the time increment for each specific cut were averaged to estimate TWA respirable dust mass concentration for that cut.

To estimate the particle size and mineralogy distributions of the respirable dust, one PC filter sample collected in the CM return location during each cut was analyzed by scanning electron microscopy with energy dispersive X-ray spectroscopy (SEM-EDX). (Notably, while PC filters were also collected in the intake location, these generally did not have sufficient particle loading for analysis due to the short sampling times.) The sample preparation and analytical procedure has been previously described in detail (see Sarver et al. 2021). Briefly, a stainless-steel trephine was used to take a 9-mm subsection of each PC filter selected for analysis. The subsection was mounted on an aluminum stub, and sputter coated with Au/Pd to render it conductive. The SEM-EDX analysis was conducted using a FEI Quanta 600 FEG Environmental SEM (Hillsboro, OR, USA) equipped with a backscatter electron detector and a Bruker Quantax 400 EDX spectroscope (Ewing, NJ, USA). Bruker’s Espirit software was used to run a computer-controlled routine (originally developed by Johann-Essex et al. (2017a, b) to identify and characterize supra-micron particles (lengths between about 1–10 μm). The routine uses image contrast to identify particles, and then it automatically collects size (length and width) and EDX data from each particle. The routine was run at 1,000× magnification and proceeded through a series of pre-programmed field locations. Then, up to 50 particles were analyzed per field and the routine proceeded until at least 500 particles had been analyzed.

Particle size distributions on each sample were determined on the basis of projected area diameter (PAD, which is effectively the average of particle length and width). To describe mineralogy, the EDX data was used to classify each particle per the criteria published by Sarver et al. (2021). The criteria are based on normalized atomic percentages of eight elements (carbon, oxygen, silicon, aluminum, calcium, magnesium, iron, and titanium), and were used to bin particles into one of the following classes: carbonaceous (C), mixed carbonaceous (MC), silica (S), silicates (including aluminum silicates, AS, and other silicates, SLO), carbonates (CB) and heavy minerals (HM). Particles that did not fit one of these classes were binned as “others” (O). In general, C and MC particles are typically interpreted as coal dust. In many mines, the CB particles are likely sourced from the application of inert rock dusting products. Notably, the geologic strata in Mine 27 (coal or rock) were not known to have substantial carbonate content. Other predominant minerals, including silica and silicates, are generally associated with the rock strata.

## Results and discussion

### Respirable dust concentrations

Table 3 summarizes the TWA respirable dust concentrations for both the Phase 1 and Phase 2 tests. As expected, concentrations were consistently higher in the CM return than in the intake location. Comparing the results for each specific cut, the return concentration was between 2.5 and 180× higher than the intake concentration.

**Table 3:** TWA respirable dust concentrations for phase 1 and phase 2 tests

Phase	Test			Cut sequence	TWA concentration (mg/m^3^)		Return/Intake Concentration	Return Top/Bottom Concentration
	No.	Variable	Condition		Intake	Return		
1	1a	FBS/vent	off/exhausting	bottom	0.146	1.181	8.1	4.5
				top	0.157	5.296	34	
	1b	FBS/vent	on/blowing	bottom	0.152	0.683	4.5	3.3
				top	0.091	2.245	25	
	2a	FBS/vent	off/exhausting	bottom	0.136	1.246	9.2	2.1
				top	0.09	2.618	29	
	2b*	FBS/vent	on/blowing	bottom	0.091	0.664	7.3	2.6
				top	-	1.7	-	
	3a	FBS/vent	off/exhausting	bottom	0.146	0.679	4.7	6.9
				top	0.22	4.714	21	
	3b	FBS/vent	on/blowing	bottom	0.261	0.654	2.5	3.0
				top	0.19	1.963	10	
2	4a	CM sprays	min P&V	bottom	0.173	0.466	2.7	26
				top	0.155	12.187	79	
	4b	CM sprays	max P&V	bottom	0.08	0.53	6.6	9.2
				top	0.12	4.902	41	
	5a	CM sprays	min P&V	bottom	0.382	1.22	3.2	3.8
				top	0.31	4.655	15	
	5b	CM sprays	max P&V	bottom	0.212	0.52	2.5	7.7
				top	0.245	3.997	16	
	6a	CM sprays	min P&V	bottom	0.03	0.528	18	9.7
				top	0.029	5.108	180	
	6b	CM sprays	max P&V	bottom	0.038	0.422	11	5.4
				top	0.025	2.285	91	

* Intake TWA concentration during top cuts in test 2b was not recorded because the pDr inadvertently switched off

Based on the CM return sampling, the data in Table 3 shows that the top cuts generated respirable dust concentrations that were 2.1–26× higher than their respective bottom cut. A paired t-test showed the difference was significant at 95% confidence (i.e., *P* = 0.0013, assuming unequal variance and two-tailed distribution). As reviewed above, previous research has suggested that CM cutting in rock should generate substantially more respirable dust than cutting in coal (Jaramillo et al. 2022; Johann-Essex et al. 2017a, b; Sarver et al. 2019, 2021). However, the novel ‘bottom, then top cut’ experimental design in the current study enables a direct demonstration of this trend. In Table 3, there are six top and bottom cut pairs having the same dust control conditions (i.e., Tests 1a, 2a, 3a and 4a, 5a, and 6a were conducted with the FBS off and exhausting ventilation, and with the minimum required water spray pressure and volume). For these cut pairs, the range of the ratio of top cut to bottom cut dust concentration was observed to be somewhat higher during Phase 2 (3.8–26×) than Phase 1 (2.1–6.9×), but no statistically significant difference was detected. The results could be related to the specific geology encountered in the different entries where the CM was operating during this study.

For the different dust control conditions tested, Table 3 shows that for both the bottom and top cuts, the dust concentration in the CM return was always observed to be lower during the FBS on and blowing ventilation conditions (Tests 1b, 2b, 3b) than during the FBS off and exhausting ventilation conditions (Tests 1a, 2a, 3a). Across all three tests, this amounted to a mean reduction in dust concentration of about 31% for the bottom cuts and about 50% for the top cuts. However, based on paired t-tests, these differences were not statistically significant at 95% confidence (i.e., *P* = 0.17 for bottom cuts, *P* = 0.08 for top cuts), which is attributed to the relatively small number of tests conducted for this study (i.e., *n* = 3 for each cut by dust control condition). Nevertheless, the observed trends are consistent with findings by Xu et al. (1991), which indicated that using FBS on and blowing ventilation led to a 13%–40% reduction in respirable dust versus FBS off and exhausting ventilation. Moreover, numerous other studies have shown that FBSs generally reduce respirable dust concentrations around the CM (Animah et al. 2023, 2024; Colinet et al. 1990, 1994, 2013; Colinet and Jankowski 1996; Fields et al. 1990; Gonzalez et al. 2022; Janisko et al. 2015; Jayaraman et al. 1987, 1990, 1992; Organiscak et al. 2016; Patts et al. 2016). The relatively higher reduction in dust concentrations observed for the top versus bottom cuts with the FBS on and blowing ventilation might be related to the relatively large difference in dust concentrations generated from the top versus bottom cuts (i.e., the FBS may be more efficient when dust concentration moving through the system is higher). Additionally, there may be effects of specific particle characteristics or flow conditions. It is noted that, while standard CMDPSU samplers were used in this field study to mimic typical respirable dust sampling procedures, isokinetic sampling would allow truer comparisons between tests where airflow varies substantially.

Regarding the effect of water spray pressure and volume, Table 3 shows that dust concentrations in the CM return were typically lower with maximum pressure and volume (Tests 4b, 5b, 6b) versus the minimum required pressure and volume (Tests 4a, 5a, 6a). The mean reduction was 21% for bottom cuts and 43% for top cuts. However, paired t-tests did not indicate statistically significant differences at 95% confidence (i.e., *P* = 0.40 for bottom cuts, *P* = 0.21 for top cuts). Notably, during the bottom cut in Test 5a, it was observed that limiting the CM cutting at the coal-roof rock interface was more challenging than in other tests, and the CM was often cutting somewhat above the visual interface. This may explain the somewhat higher dust concentration associated with the bottom cut and somewhat lower concentration associated with the top cut in Test 5a (versus Tests 4a and 6a). As noted above, prior studies on the impact of increased spray pressure and volume have yielded inconsistent results (Beck et al. 2018; Colinet et al. 1991; Jayaraman et al. 1984; Jiang et al. 2022, 2023; Pollock and Organiscak 2007; Schroeder et al. 1986). In practice, however, this strategy is often considered when additional dust control is needed at the CM face. The results of the current study suggest that, under the conditions in Mine 27, increasing spray pressure and volume could further reduce respirable dust concentrations downwind of the CM, with the most significant impact likely on dust generated from the roof rock. While the mechanisms (e.g., material wetting versus particle capture) and key factors contributing to this enhanced dust control (e.g., spray orientation) are outside the scope of the current work, they are deserving of additional study.

### Particle size and mineralogy

**Fig. 4:**
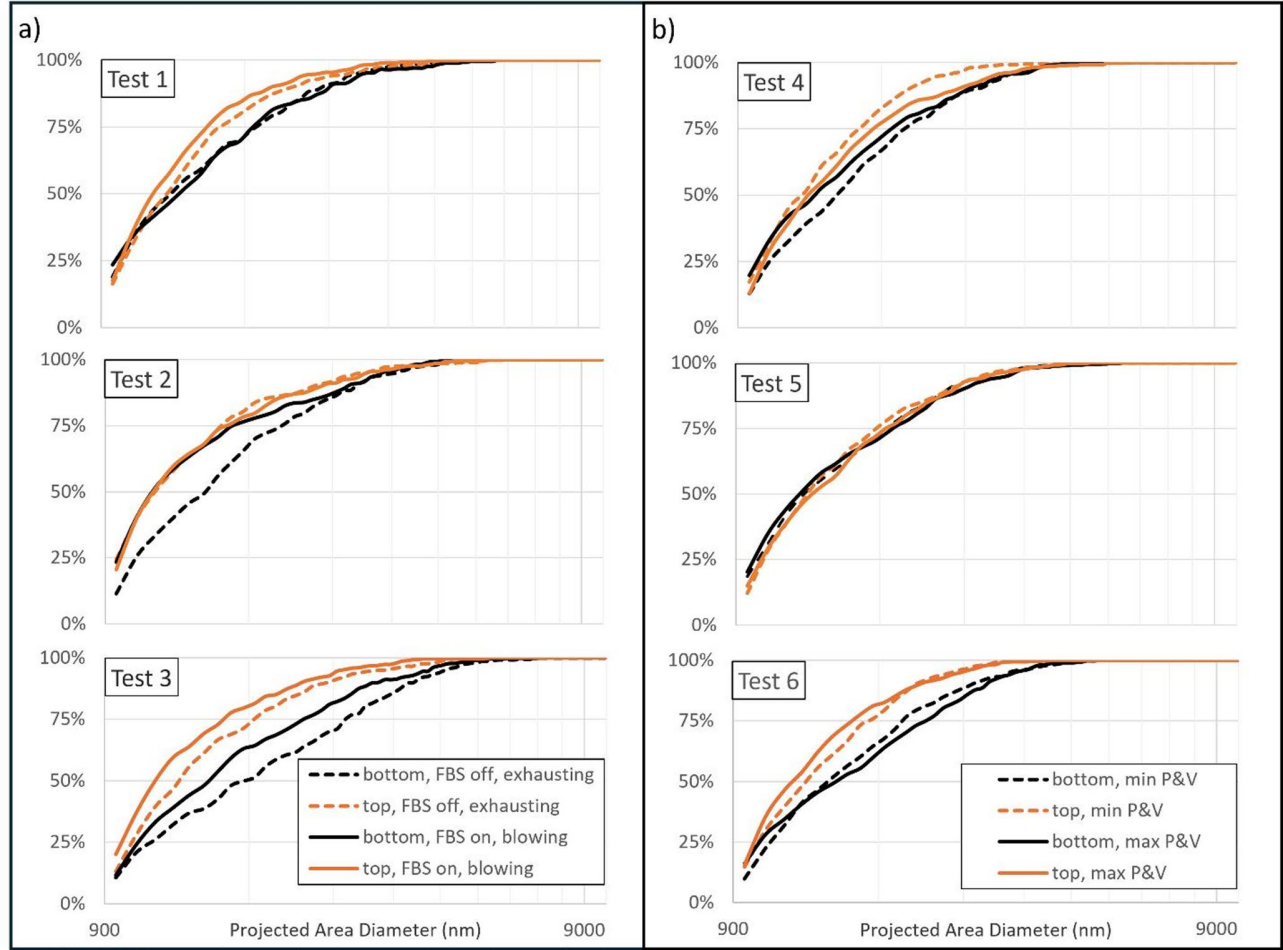
Respirable dust particle size distributions based on SEM-EDX analysis for **a** Phase 1 and **b** Phase 2 tests. The plots show the cumulative number percentage of particles that are finer than a given size

Figure 4 summarizes the particle size data derived from the SEM-EDX analysis on PC filter samples collected in the return location during each CM cut. The plots show cumulative size distribution based on the projected area diameter. In general, the dust generated from the top cuts was observed to be somewhat finer than the dust generated from the bottom cuts. This is consistent with expectations from a few prior studies (Organiscak et al. 1990; Ramani et al. 1987), and is likely related to differences in material hardness between the roof rock targeted by the top cuts and the coal strata that made up most of the bottom cuts. Notably, very little difference in particle size distribution is seen for Test 5. This could be related to the tendency of the CM cutting to cross further into the roof rock strata during the bottom cuts for this test, which also fits with the observation of minimal change in the dust concentration generated from the top cut when the spray condition was changed for this test (Table 3).

For the Phase 1 tests, the FBS and ventilation conditions may have had some effect on the particle size distribution. Comparing the bottom cuts in Test 2 and the top and bottom cuts in Test 3, dust was generally finer under the FBS on and blowing ventilation conditions than under FBS off and exhausting ventilation conditions. A similar but less pronounced effect is also evident for the top cuts in Test 1. This is consistent with the results of prior studies indicating that FBSs have higher removal efficiency for relatively coarse particles (Animah et al. 2023, 2024). On the other hand, for the bottom cuts in Test 1 and top cuts in Test 2, the FBS and ventilation conditions do not appear to influence the particle size distributions. This is probably related to the fact that the bottom cut particle size distribution was already quite fine in Test 1 (and the FBS is more efficient on coarser particles), and the relatively low dust concentration generated from the top cut in Test 2 (i.e., compared to all other tests without the FBS operating). In Phase 2, no consistent trends in particle size are apparent with the increase in CM spray pressure and volume.

**Fig. 5:**
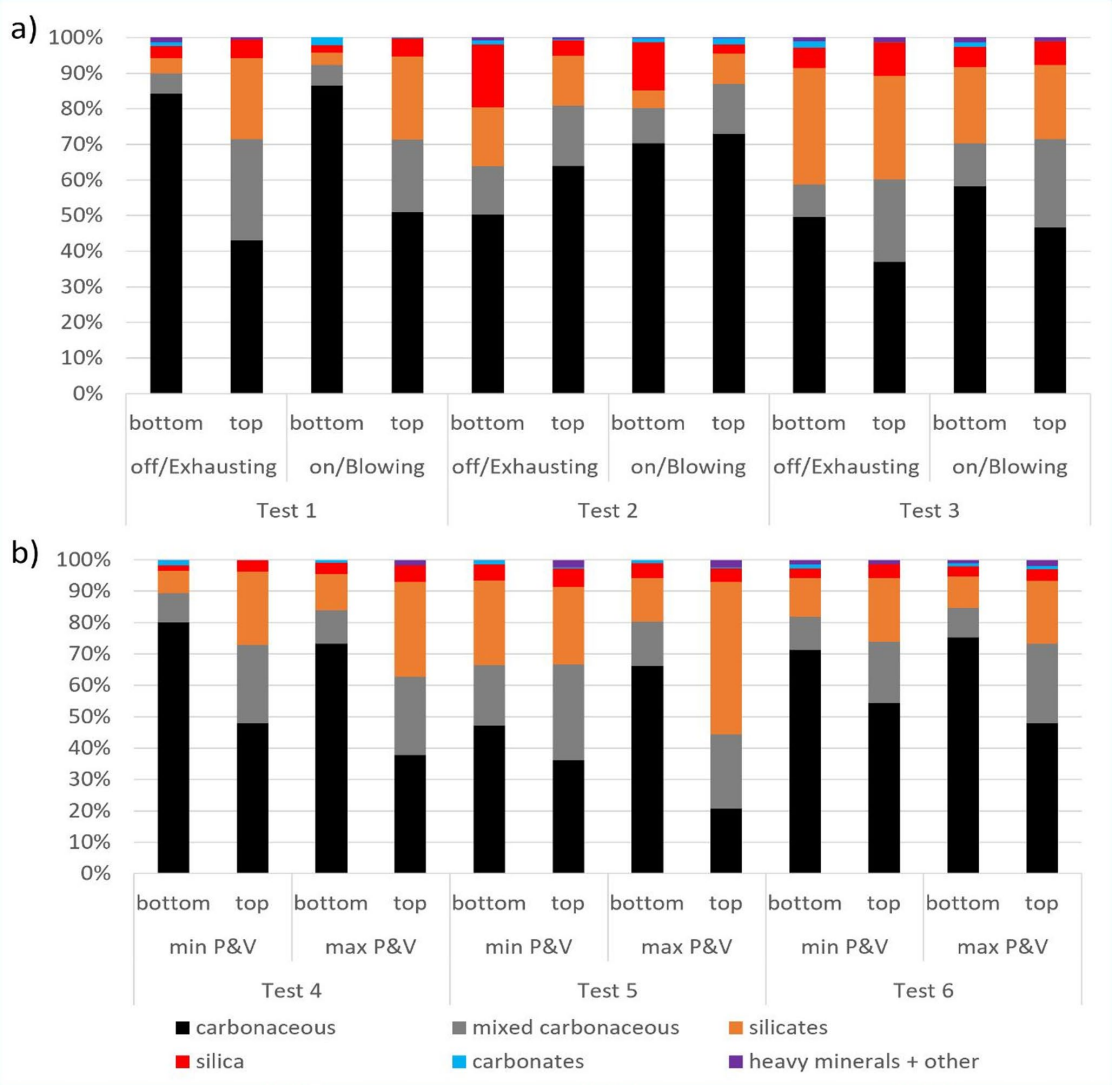
Mineralogy distribution of respirable dust particles by number percentage based on SEM-EDX analysis for **a** Phase 1 and **b** Phase 2 tests

Based on the SEM-EDX analysis of PC filter samples collected in the return location during each CM cut, Fig. 5 presents the distribution of respirable dust particles by primary mineralogy classes. The plots show the mineralogy distribution based on particle number. Notably, the carbonaceous particles were observed to be somewhat finer than mineral particles (data not shown). Therefore, if the results in Fig. 5 were used to estimate mineralogy distributions on the basis of mass, carbonaceous content would appear somewhat lower whereas mineral contents would appear somewhat higher. The minimal abundance of carbonate particles suggests that rock dusting products were not a primary source of respirable dust in the CM return sampling location during this study.

Figure 5 shows that respirable dust generated from bottom cuts generally included more carbonaceous particles whereas dust generated from top cuts included more silica and silicate particles. (Test 2 is a notable exception, and this could be related to the specific geology in the entry and relative size distribution of different particle types.) Overall, however, the dust composition was considerably mixed for most cuts—despite the targeted “bottom, then top” cut sequence used for this study. Mixed dust is not completely unexpected since the coal and roof rock strata should undoubtedly have impurities (Jaramillo et al. 2022). Furthermore, the interface between these strata is imperfect and could be thought of more as a transition zone. As mentioned, in Test 5a, the CM was observed to cut somewhat above the visual interface between the coal and roof rock during the bottom cut. Indeed, the relative abundance of mineral particles from this bottom cut was the highest of all bottom cuts in the Phase 2 tests. Additionally, it should be reiterated that some floor rock was taken with the coal during many of the bottom cuts (per Fig. 1). Nevertheless, these results clearly demonstrate that, even when CM cutting is aimed primarily at the coal seam, the respirable dust produced can contain substantial mineral content, including silica and silicates.

Figure 5 reveals no consistent effects of changing CM spray pressure and volume on the respirable dust composition. For the FBS and ventilation conditions, the results are nuanced. Dust sampled in the CM return under FBS on and blowing ventilation (versus FBS off and exhausting ventilation) exhibited a lower relative ratio of mineral to carbonaceous particles for Tests 2 and 3. However, this trend is not observed for Test 1, for which the dust particle size and mineralogy distributions appear similar between the bottom or top cuts regardless of the FBS and ventilation condition. While field data on the relative effects of FBSs on different dust constituents is scarce, early laboratory studies by Colinet et al. (1990) suggested that FBSs may be more efficient for the reduction of coal dust versus silica. At least under the conditions tested in Mine 27, no such effect is evident here. Rather, the samples collected while the FBS was operating (Tests 1b, 2b, 3b) generally had slightly less silica (by both number and mass percentage) than their paired samples while the FBS was not operating (Tests 1a, 2a, 3a). However, paired t-tests indicate that the difference is not significant at 95% confidence for either the bottom cuts (*P* = 0.32 or *P* = 0.80 based on number or mass percentage, respectively) or the top cuts (*P* = 0.23 or *P* = 0.13 based on number or mass percentage, respectively).

## Study implications and conclusions

Previous studies by the authors’ research team have concluded that CM cutting into rock strata can generate an inordinate amount of respirable dust compared to cutting in coal (Jaramillo et al. 2022; Johann-Essex et al. 2017a; Sarver et al. 2019, 2021). However, those studies relied on dust samples that represented the entire mining height in a typical entry, including both the target coal seam and any roof, floor, or interburden rock that would normally be cut at the production face. The novel “bottom, then top” CM cutting sequence employed for the current study enables more direct insights regarding the concentration and characteristics of dust generated from rock versus coal strata. Results clearly show that CM cutting in rock generates much more respirable dust. As mentioned, top cuts (mostly in roof rock) yielded dust concentrations that were 2.1–26× higher than their respective bottom cuts (mostly in coal), and these findings are even more remarkable considering the relative strata heights being cut. Here, the bottom cut heights were about 2.2–2.9× greater than the top cut heights, which translates to a similar difference in the volume of material extracted between the bottom and top cuts since the cut depth remained constant. This indicates that CM cutting in rock generated 4.6–75× more respirable dust than cutting in coal on a unit-height basis.

The observed differences in particle size between respirable dust generated from top and bottom cuts may also have important implications. Particle size is well-established as an important factor in terms of lung dust deposition, mobility, and reactivity (Assemi et al. 2023; Fan and Liu 2021; Mischler et al. 2016; Pan et al. 2021; Salinas et al. 2022; Shekarian et al. 2021; Zhang et al. 2021, 2022). Moreover, observations of mixed dust composition despite efforts to target primarily coal versus rock strata are noteworthy. While cutting rock can generate higher concentrations of respirable dust, even selective mining to stay within the coal seam can generate dust with substantial mineral content. Limiting respirable silica concentrations, in particular, has been and will continue to be a major focus in coal mines (MSHA 2024).

CM-mounted FBSs are a common piece of the overall dust control strategy in many room and pillar mines. The results presented here suggested that, under blowing ventilation conditions, FBSs can be particularly effective at reducing respirable dust concentrations associated with roof rock cutting. To enhance dust suppression, increased water spray pressure and volume have often been considered. For the CM sprays tested in Mine 27, results suggest this approach may have some merit in reducing dust concentration, and perhaps more so for dust generated from the harder strata (i.e., rock). Especially for mines that have the capacity to increase spray pressures and volumes, or that could experiment with sprays generating finer droplets or reoriented sprays to target dust coming from the roof strata, engineering studies should be conducted to evaluate such modifications.

Overall, the research reported here provides practical evidence of the very different respirable dust-generating potential that exists between adjacent geological strata when cut by a CM. Nevertheless, the limitations of this work should be duly acknowledged. This study was conducted in just two sections of a single mine and with a relatively limited set of samples per condition. Considering the variability in geology and strata characteristics (heights, hardness, specific mineralogy) even within a single mine section, the specific results presented here might not be widely generalizable. However, the main trends are informative, and the experimental design should be applied in other mines or to test additional conditions. Moreover, future research studies should focus on investigating the mechanisms of dust generation and control by quantifying the geotechnical properties of the relevant mine strata and the key variables associated with dust capture (e.g., water spray droplet size and concentration). Such work would support the design and adoption of CM cutting bits that maximize coal production while reducing respirable dust generation or water spray nozzle selection and operating conditions that are aligned to the specific dust profile of a mine.

## Data Availability

All data is available within the article or upon reasonable request from the authors.
